# Encryption in phase space for classical coherent optical communications

**DOI:** 10.1038/s41598-023-39621-5

**Published:** 2023-08-10

**Authors:** Adrian Chan, Mostafa Khalil, Kh Arif Shahriar, David V. Plant, Lawrence R. Chen, Randy Kuang

**Affiliations:** 1 Quantropi Inc., Ottawa, ON K1Z 8P8 Canada; 2 Department of Electrical and Computer Engineering, McGill University, Montreal, QC H3A 0E9 Canada

**Keywords:** Engineering, Optics and photonics

## Abstract

Optical layer attacks on optical fiber communication networks are one of the weakest reinforced areas of the network, allowing attackers to overcome security software or firewalls when proper safeguards are not put into place. Encrypting data using a random phase mask is a simple yet effective way to bolster the data security at the physical layer. Since the interactions of the random phases used for such encryption heavily depend on system properties like data rate, modulation format, distance, degree of phase randomness, laser properties, etc., it is important to determine the optimum operating conditions for different scenarios. In this work, assuming that the transmitter and the receiver have a secret pre-shared key, we present a theoretical study of security in such a system through mutual information analysis. Next, we determine operating conditions which ensure security for 4-PSK, 16-PSK, and 128-QAM formats through numerical simulation. Moreover, we provide an experimental demonstration of the system using 16-QAM modulation. We then use numerical simulation to verify the efficacy of the encryption and study two preventative measures for different modulation formats which will prevent an eavesdropper from obtaining any data. The results demonstrate that the system is secure against a tapping attack if an attacker has no information of the phase modulator and pre-shared key.

## Introduction

With increasing demand for faster, more affordable, and smaller form factor solutions in optical communications, the security of the optical network becomes essential in protecting the immense amount of information that is transmitted. Currently, optical networks are mainly secured by protocols at the second layer of the OSI model and above, relying on a software-based solution to secure communication^1^. However, security threats at the physical and optical layer cannot be ignored as eavesdroppers can have unfettered access to the optical layer and potentially compromise data transmitted. With the major development in recent years with quantum computers, ciphertexts are potentially vulnerable and can be cracked in significantly shorter periods of time. By utilizing an optical layer encryption, security can be increased and resist attacks at these layers. Applying an optical layer encryption in the system will allow for low latency, protocol agnostic, enhanced security and transparent end-to-end communication can be achieved.

Various methods have been proposed and implemented for enhanced security in the physical layer of the system including optical code division multiple access (O-CDMA)^2^, optical chaos signal generation^3^, optical steganography^4^, and XOR encryption^5^. These methods have shown vulnerabilities against attacks such as optical chaos signal generation and optical steganography being vulnerable to time-delay identification and post-processing statistical methods. The security performance of O-CDMA has been investigated thoroughly but remains an open issue and is highly dependent on system design and implementation^6^. Similarly, alternative secure communication systems have been presented for key distribution using quantum mechanics such as Quantum Key Distribution (QKD) which is based on the theoretically secure BB84 protocol^7^. One common layout is continuous-variable QKD (CV-QKD) which is based on using an amplitude such that the on-state is at a level that prevents an eavesdropper, Eve, from discriminating the signal and allowing Alice and Bob to detect an eavesdropper by comparing measurements and the variance of the distribution^8^. Gaussian Modulated Coherent State CV-QKD has demonstrated its capability of reaching a secure key rate of 7.04 Mbps over 25 km of fiber^9^. These systems can be used in parallel with conventional data communications through Dense Wavelength Division Multiplexing (DWDM) technique to achieve both data transmission and secure key distribution^10^. Another common and more recent layout is twin-field QKD (TF-QKD)^11^ which can overcome the PLOB-repeaterless bound^12^. Recently, this system has demonstrated its capability of reaching over 800 km at a secret key rate of 0.014 bps by utilizing a four-phase twin-field protocol and high-quality set-up^13^. These systems, although scientifically secure with its proven security against general attacks and information-theoretic security, are not recommended by major entities such as the National Security Agency (NSA)^14^. This is because security models are unable to encompass all features of a real-world component in preparation and detection, and can only provide a guidance where each specific set-up must undergo a thorough study^15^. More work must be accomplished in this field before it can be adapted for commercial use. Nevertheless, QKD, on its own, is only capable of distributing keys used for digital data encryption and its current limitations prevent it from being used for direct quantum encryption for high-speed coherent optical transmission^10,16^.

An alternative approach is to use an encryption scheme where high speed classical equipment is used to generate a noisy signal which masks the original data. But since such encryptions in classical systems lack mathematically provable security, it is extremely important to design the encryption scheme such that any attempt to eavesdrop on the system would fail under reasonable and practical assumptions.

Here, we present the design, simulation, security analysis, and preliminary experimental results of an optical layer security design that imposes a mask of noisy phases on the data to make it obscure for an eavesdropper. From here on, we refer to this as Encryption in Phase Space (EPS). EPS exploits phase-shifting operators to encrypt the optical signals. EPS is a symmetric encryption scheme which is adapted for classical communication system from the asymmetric encryption presented by Kuang and Bettenburg in 2020^17^, also known as Coherent-based Two-Field QKD (CTF-QKD), which was developed as an alternative to the QKD protocol by utilizing a public key envelope. EPS is intended to be used after key distribution between Alice and Bob using techniques such as CTF-QKD. Therefore, asymmetric encryption will be used to establish the shared key while the symmetric encryption scheme will be used for data encryption after the secret key has been shared between the two users.

In terms of the infrastructure of the system, EPS is also similar to PSK-Y00^18^ and QAM-QNSC (Quantum Noise Stream Cipher)^19^ with the exception that these encryption schemes rely on the quantum noise existing in the continuous light of a laser and hence operate as a quantum system whereas EPS uses classical equipment commercially used in high-speed networks to generate the noise required for encryption. EPS can be regarded as a general case of PSK-Y00 or QAM-QNSC. PSK-Y00 uses equal sliced basis phases driven by the PSK, whereas EPS uses truly random phases not limited to equally sliced phases. In PSK-Y00 or QAM-QNSC, phase and/or amplitude modulation of coherent light is used as the base of signal transmission and encryption on it is performed by using phase fluctuation (quantum fluctuation) of light^18,19^. First, legitimate users share a secret key and use it to generate a pseudo-random bitstream using the same pseudo-random number generator (PRNG). Next, they perform conventional optical communications in which additional noise arising from the uncertainty principle of the electromagnetic field itself is used to further mask the data. This additional noise is a theoretical consequence of the laser light theory by Glauber and Sudarshan^20,21^. Hence, the Y-00 protocol combines mathematical noise encryption (through the secret shared key and PRNG) and physical noise encryption (using quantum fluctuations of light). On the other hand, EPS does not use physical quantum shot noise for achieving security. The two legitimate users share a secret key generated by a PRNG which is used to drive a phase modulator to generate the phase noise to mask the data. Like CTF-QKD, EPS generates an envelope based on the pre-shared secret, then performs a standard modulation scheme to encode data at the transmission side. The authorized receiver will then remove the envelope based on the same pre-shared secret before coherent detection or through digital signal processing (DSP) after detection. This allows the transmission to be performed in one-direction, from Alice to Bob, to maintain the confidentiality and integrity of the system. Confidentiality and integrity are maintained by leveraging a deterministic pseudorandom number generator (PRNG) driven phase encoding to generate an envelope at Alice transmission (Tx) to encrypt the data that she will send to Bob receiver (Rx). Generating an envelope will provide security in an existing infrastructure while having minimal impact on the performance of the optical communication system. The security of this system follows the same encryption that has been described in detail in^22^, where an attacker will obtain nondeterministic results from an invisible tap while Alice and Bob are able to operate deterministically. This contrasts with QKD where Alice and Bob are not able to perform normal operation while Bob is able to detect attacks. While the principle of using such mathematical encryption is not new, this work shows that by choosing appropriate system and signal parameters, it is possible to prevent eavesdropping using mathematical encryption only for the envelope while allowing the legitimate receiver to decode the data from the envelop. In other words, this work specifies the optimum operating conditions which prevent eavesdropping while allowing the authorized receiver to unmask the data.

## Methods

### Encryption in phase space (EPS) and security analysis

EPS is based on applying phase-shifting operators to coherent states. A phase-shifting operator is applied to the coherent state at the transmitter and is shown as,

1 $$\begin{array}{c}\widehat{U}\left(\varphi \left(t\right)\right)\left|\alpha \right.\rangle =\left|\alpha {e}^{i\varphi \left(t\right)}\right.\rangle ,\end{array}$$

where *Û(φ(t))* is the phase-shifting operator driven by a PRNG seeded with a pre-shared key.

Leveraging the fact that phase-shifting operators are unitary, the conjugate transpose can be applied to reverse the phase shifted operation at the receiver by using the same PRNG with the pre-shared key between both the transmitter and receiver operator,

2 $$ \hat{U}\left( {\varphi \left( t \right)} \right)^{\dag } \hat{U}\left( {\varphi \left( t \right)} \right)|\left. \alpha \right\rangle = |\left. \alpha \right\rangle $$

By applying the operator’s conjugate transpose, the identity operation is obtained for $\hat{U}\left( {\varphi \left( t \right)} \right)^{\dag } \hat{U}\left( {\varphi \left( t \right)} \right) = \hat{I}$ since *Û(φ(t))* is a unitary operator. Therefore, the original coherent state can be recovered. In the aspect of our technology, the phase-shifting operator and conjugate transpose operator can be applied by a PM or in DSP at the receiver.

The basis of EPS can be related to the operation of CV-QKD. CV-QKD operates by the quadrature of the electric field in the optical phase space by transmitting coherent states from Bob to Alice by randomly selecting between the “off” ($\left|0\right.\rangle$) or “on” state ($\left|1\right.\rangle$). The random selection is typically achieved by modulating the phase to impose the $\left|0\right.\rangle$ with 0 degrees or $\left|1\right.\rangle$ state with 180 degrees. The amplitude is tuned to a level where the probability distributions of both states overlap. The variance from the measurement will be used to determine whether tampering occurs. In contrast to CV-QKD where the global reference phase is static, EPS leverages the phase space where the reference space of the in-phase and quadrature operator of the coherent state is manipulated with the phase-shifting operator, making the global reference space dynamic. This dynamically changes in time through phase modulation, which is driven by a PRNG, and decryption can easily be accomplished through a pre-shared key. That is, for EPS, the “static” global phase can only be established between the trusted transmitter and receiver with the pre-shared key. In detection, if the expected BER increases then it can be determined that an eavesdropper was present. Furthermore, even with an “invisible” tap with little disruptions on the trusted communications, the encryption will result in the eavesdropper obtaining random data with a high BER around 0.5 or leaving the maximum uncertainty to the eavesdropper.

This EPS will secure the optical line by applying different phase shifts to the coherent state preventing an eavesdropper from obtaining any information of the coherent state, creating a noncoherent channel. This is achieved when an eavesdropper taps the fiber. The tapped signal that an eavesdropper will have to decrypt is $\left|\alpha {e}^{i\varphi (t)}\right.\rangle$, where the coherent state is masked by the phase shifted operation. With a time-varying phase shift, the coherent state will remain random, masked, and secure against an attack. Additionally, in practice, there will be differences between the receiver’s Local Oscillator (LO) and the eavesdropper’s LO, which will increase measurement error. Therefore, it is more exact to describe the tapped signal that an eavesdropper will have to decrypt as $\left|\alpha {e}^{i(\varphi \left(t\right)+\Delta {\varphi }_{p}+\Delta {\varphi }_{LO})}\right.\rangle$.

It is also important to touch on the generator that will determine *φ(t)*. *φ(t)* can be driven by a PRNG component such as the deterministic Pseudo-Quantum RNG (PQRNG). The deterministic PRNG unit must have good randomness to prevent correlation of future values and a long secret to increase the difficulty of decoding. One class of PRNGs that fit these requirements is PQRNG such as the one described in^23^. The PQRNGs in^23^ can be generated by supplying random numbers to select specific permutation matrices in the quantum permutation pad. Utilizing the novel quantum permutation pads, the PQRNG is capable of holding over 100,000 bits of entropy with 64 8-bit permutation matrices through the pre-shared secret to deterministically drive the phase-shifting operator, providing added security to the system. The entropy of this PQRNG can quickly be scaled to increase the security by increasing the bits of the system and number of permutation matrices. The PQRNG is seeded with a pre-shared secret of up to 16 kB supplied by a telco operator. This method will prevent an eavesdropper from decrypting the phase randomization both physically and digitally and maintain the confidentiality of the data over the fiber optical layer. Other PRNGs such as a genuine QRNG can be used, however require a large set of random numbers pre-shared between Alice and Bob and used repeatedly between a synchronized EPS encryption and decryption. Another alternative to apply a genuine QRNG is to send the generated values over another channel to drive the encryption, similar to chaotic phase scrambling^24^. Deterministic PRNGs are used in this study to simplify the synchronization between encryption and decryption.

Finally, EPS’s security can be described through the calculation of mutual information similar to a noncoherent channel where the phase information is unable to be transmitted over the channel. The description below will extend the work performed in^25^ with the addition of our EPS implementation. The EPS system will be assumed to follow a noncoherent channel as the phases will be completely randomized through a uniformly distributed deterministic PRNG. Noncoherent channels can be used to model EPS as they are AWGN channels which have introduced random phase rotations^26^. The random phase rotations in the EPS case are a result of the phase-shifting operators applied onto the optical signal. Randomization is achieved through the phase modulator (PM) driven by a PRNG. This noncoherent channel will provide a lower bound of information leakage that Eve will be able to obtain in the ideal situation. Indeed, a complete analysis will be performed in the future to identify realistic cases with limited number of phase slices, maximum phase shifts applied, and limiting the tapping power. Without the encryption, data travels through a coherent channel containing information from phase or amplitude or both, depending on the modulation format. Based on the SNR, a malicious party can obtain information and will be explained from the following model. The channel will have a complex-valued input,

3 $$\begin{array}{c}X={X}_{\parallel }\cdot {e}^{j{X}_{\sphericalangle }}, {X}_{\parallel }\epsilon \left[0,\infty \right), {X}_{\sphericalangle }\in \left[-\pi ,\pi \right),\end{array}$$

and a complex-valued output,

4 $$\begin{array}{c}Y={Y}_{\parallel }\cdot {e}^{j{Y}_{\sphericalangle }}, {Y}_{\parallel }\epsilon \left[0,\infty \right), {Y}_{\sphericalangle }\in \left[-\pi ,\pi \right).\end{array}$$

For a partially coherent the continuous-time form can be described by,

5 $$\begin{array}{c}Y\left(t\right)=X\left(t\right)\cdot {e}^{j\Theta \left(\mathrm{t}\right)}\cdot {e}^{j\varphi \left(t\right)}+N\left(t\right),\end{array}$$

where *ϴ(t)* is the phase noise process, *φ(t)* is the phase space randomization and *N(t)* is the complex-valued additive white gaussian noise (AWGN) process with a variance of *2σ*_*n*_^*2*^. An ideal interleaver and de-interleaver can convert (3) into the discrete-time form following the form,

6 $$ \begin{aligned}   Y_{i}   & = \left( {X_{i}  + N_{i} } \right) \cdot e^{{j\Theta _{i} }}  \cdot e^{{j\varphi _{i} }}  \\ &  = X_{i}  \cdot e^{{j(\Theta _{{\text{i}}}  + \varphi _{i} )}}  + N_{i} \prime , \\  \end{aligned}  $$

where $${N}_{i}{\prime}\sim {N}_{\mathbb{C}}\left(0, 2{\sigma }_{n}^{2}\right)$$.

Polar decomposition of mutual information for an AWGN channel with Gaussian input, phase noise, and encryption can be calculated through the equations presented in^25^. The mutual information *I(X;Y)* is described as,

7 $$\begin{array}{c}I\left(X;Y\right)=\\ I\left({X}_{\parallel };{Y}_{\parallel }\right)+I\left({X}_{\sphericalangle };{Y}_{\sphericalangle }|{X}_{\parallel }\right)+I\left({X}_{\parallel };{Y}_{\sphericalangle }|{Y}_{\parallel }\right)+I\left({X}_{\sphericalangle };{Y}_{\parallel }|{X}_{\parallel },{Y}_{\sphericalangle }\right)\end{array}$$

where the terms on the right side of the equation from left to right represents the Amplitude term, Phase term, Mixed term I, and Mixed term II. Mixed term I is the amount of information about the input amplitude that can be drawn from the output phase given the output amplitude. Mixed term II is the amount of information about the input phase that can be observed from the output amplitude given the input amplitude and output phase. This polar decomposition represents the information that is sent in each component and can be related to the total amount of information that attainable by the receiving party or a malicious party in a coherent channel. The polar decomposition of the mutual information was plotted in 1(a) by Goebel et al*.* for 16-QAM^25^.

**Figure 1:**
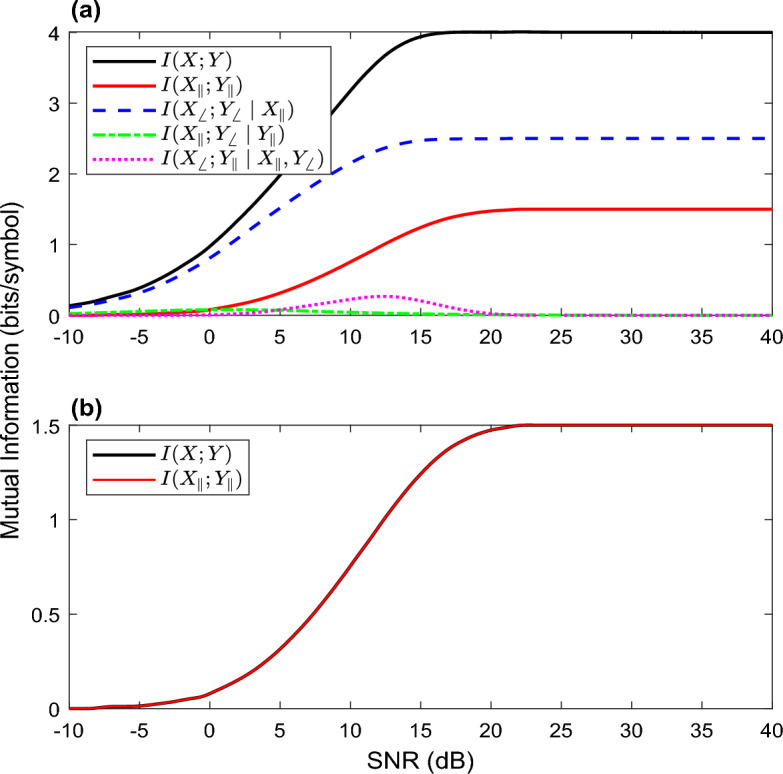
Polar decomposition of mutual information for (**a**) 16-QAM^21^ and (**b**) 16-QAM with EPS applied.

EPS can easily be integrated for standard data modulation formats. As the phase is completely randomized through the pseudo-random selection of phases and is uniformly distributed, the output phase y_∢_ will contain no information resulting in the Phase term and Mixed term I to equal zero. Since y_∢_ carries no information, the Phase term will also have no mutual information available resulting in the Phase term to be zero. Mixed term I tends toward zero because it becomes a continuous concentric ring with an infinite number of phases. Mixed term II will also be assumed to equal zero due to $p\left({y}_{\parallel }|{x}_{\parallel },{y}_{\sphericalangle }\right)=p({y}_{\parallel }|x,{y}_{\sphericalangle })$. Therefore, the mutual information only contains the Amplitude term, *I(X; Y)* = *I(X*_‖_*; Y*_‖_*)*. Indeed, this scenario is ideal and in practice, a finite number of phase levels would be chosen where the Phase term would have some value as long as the variance is small, and the Mixed term II would have a negligible small value. This will be the mutual information that travels through the fiber and also the maximum information that Eve can obtain if she taps 100% of the power. The ideal polar decomposition of mutual information for Eve with EPS applied to 16-QAM is shown in Fig. 1b. As expected, the maximum and ideal mutual information that Eve can obtain from a noncoherent channel is significantly reduced compared to Fig. 1a and in a realistic scenario where Eve would only tap a small amount of power, the Amplitude term that she would obtain would be even smaller. On the other hand, Bob can convert the noncoherent channel back to a coherent channel by applying the decryption using the pre-shared secret and obtaining the modulation output phase y_∢_. Doing so will allow Bob to recover the Phase term, Mixed term I, and Mixed term II. Therefore, his mutual information will consist of all the mutual information terms shown in (7) and in the ideal situation will be the same as Fig. 1a once decryption is performed. These findings can also be extended to Phase Shift Keying (PSK) modulation formats where no amplitude information would be present resulting in a mutual information approaching zero in the ideal case.

To summarize, this initial mutual information study with the idea assumption and cases have provided a lower bound and guidance in the leakage of information with the conclusions listed:

1. Bob is able to recover all mutual information terms through decryption with knowledge of the pre-shared key.
2. PSK modulation formats provide the lowest amount of information leakage as the phase slices increase.
3. PAM formats provide the least security as the amplitude term remains through encryption.
4. QAM is a mixture of both amplitude and phase modulation which results some information leakage, mainly due to the amplitude modulation.
5. Eve still obtains cipher bits and is required to know the pre-shared key in order to obtain all information.

A more comprehensive study will be performed in the future to accurately quantify the security the EPS in a standalone paper. Nevertheless, the results shown in this mutual information description of EPS operating over the optical fiber demonstrates that a malicious party will only be able to obtain a minimal amount of information for QAM modulation formats and negligible amounts of information for PSK modulation formats.

### Setup for simulations and experiments

**Figure 2:**
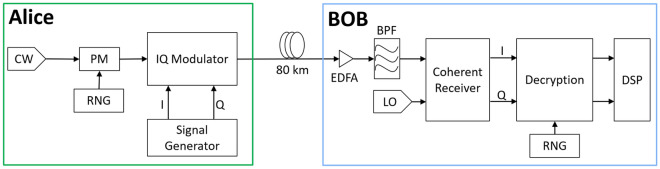
Schematic diagram of the proposed EPS system. *CW* continuous wave laser, *BPF* band-pass filter, *I* in-phasesignal, *Q* quadrature signal.

The EPS system schematic is shown in Fig. 2. There are two main sections: the transmitter, where phase encryption and data encoding occur, and receiver, where phase decryption and data decoding occur. The first section, Alice Tx, generates a coherent light which is encrypted by a PM and driven by a deterministic PRNG seeded with the pre-shared secret. This PM acts as a phase-shifting operator which randomizes the phase of each coherent state. A pre-shared key is required to allow both users to encrypt and decrypt their data. This requirement may offer telco operators the advantage to control their data security over the infrastructure layer and avoid any possible security backdoor set by optical transceivers. Alice’s data will then be encoded into the phase randomized coherent light through an IQ-MZM using the modulation of their choice. Alice’s encrypted data or optical cipher signal is then sent to Bob. Bob will receive Alice’s optical cipher signal and perform coherent detection. Decryption will be performed in DSP where the pre-shared key will be used to remove the effect of Alice’s phase-shifting operator. This step is performed by applying the conjugate transpose of the initial phase shift digitally to the detected signal. Finally, typical DSP algorithms used in coherent receivers can be performed to obtain Alice’s encoded data. In essence, the major difference between the system presented in Fig. 2 and a conventional coherent optical communication system is the addition of an optical layer encryption at Alice’s transmitter and an additional decryption step at Bob DSP.

Next, the communication from Alice to Bob is described in detail. Firstly, Alice creates a coherent state, $|{\alpha }_{b}\rangle =\left|\sqrt{\mu }\rangle \right.$, where *μ* is the signal intensity. She will then apply phase randomization to the optical signal resulting in $|{\alpha }_{b}\rangle =\left|\sqrt{\mu }{e}^{i{\varphi }_{b}}\rangle \right.$ where *φ*_*b*_ is the phase randomization applied through a PM driven by a deterministic PRNG. Furthermore, this PRNG unit will be used by both Alice and Bob together to generate the same randomized pattern to allow for seamless encryption at Alice and decryption at Bob. The phase randomized state will act as an envelope where Alice will then encode her information using their desired standard modulation format, resulting in the output coherent state $|{\alpha }_{b}{\prime}\rangle =\left|\sqrt{{\mu }_{b}}{e}^{i({\varphi }_{b}+{\varphi }_{a})}\rangle \right.$, where *φ*_*b*_ is the phase randomization and *φ*_*a*_ and ${\mu }_{b}$ represent the phase and intensity of the modulated data that Alice sends to Bob. The optical signal will then be sent to Bob, where he will perform coherent detection and obtain an optical power that is incident at the photodetectors given as,

8 $$\begin{array}{c}P=\mu b+\nu +2\sqrt{{\mu }_{b}\nu \mathrm{cos}\left({\varphi }_{a}+{\varphi }_{b}\right)} ,\end{array}$$

where ν is the LO intensity at Bob Rx. The rate of the PRNG is the same as that of the modulated signal. The signal driving the PM, i.e. mod(t), contains a block of 10^4^ symbols containing only zeros at its overhead. Hence, after the PM, for the overhead portion, ${P}_{out} (t)={P}_{in} (t)$ which is a constant amplitude CW light. When the output from the PM gets modulated by the IQ modulator, that overhead portion takes the form of the standard modulation format used by Alice while the rest of the IQ modulator output becomes the standard format signal mixed with phase noise coming from the non-overhead section of the PM output. At the receiver, before any decryption, the received signal is demodulated for its standard format. Now, only that part of the received signal remains in standard format (QPSK, 16-QAM etc.) which was mixed with the overhead block of the encryption key. As a result, when demodulation is done without decryption, only for that standard-format section of the received signal, we obtain a low BER while the rest of the demodulated signal gives a BER of 0.5. In this way, the instant of signal sample where decryption should start is identified.

In contrast to^17^, in (8), the envelope remains at the detection and will be removed digitally. This can be achieved because Bob and Alice have a pre-shared key to apply the phase-shifting operation digitally. Only Alice’s encoded information will then remain. On the receiver side, Bob first captures the data using an analog-to-digital converter (ADC). Then the captured data is then decrypted by Bob offline and the signal quality is improved through signal processing algorithms in the digital domain on a computer, offline. The data rates reported in this work denote the date rate of signal propagation through the optical fiber. Typical DSP algorithms include but are not limited to DC blocking, resampling, QI compensation, dispersion compensation, nonlinear compensation, timing recovery, adaptive equalizer, frequency offset estimation, and carrier phase estimation. For the purposes of simplicity in simulation and experiment, a single polarization was used. It should be possible to adapt the proposed method in PDM systems since the polarization of the signal should not affect the phase noise used for encryption. For each polarization, different encrypting phase noises can be used.

**Figure 3:**
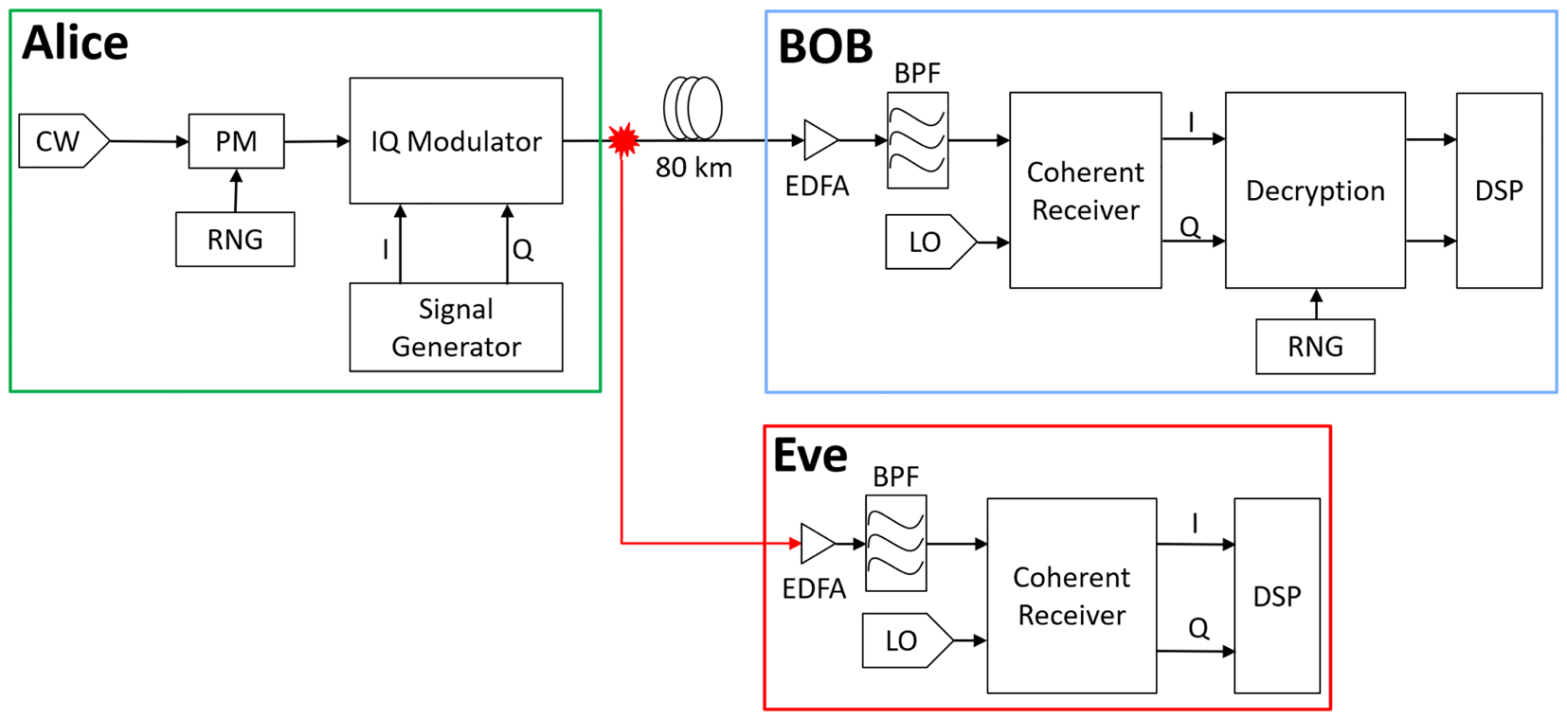
Schematic diagram of the EPS system with a tapping attack.

The system schematic of a typical attack on the optical fiber where an eavesdropper taps the EPS system is shown in Fig. 3. In essence, we will be simulating the most desirable attack by Eve where she is next to Alice and has access to her output port. An eavesdropper receiver would receive a power equation like (8). This received signal will be lower in quality and be weaker in power due to Eve only being able to tap a small portion of the transmitted power.

Single polarization simulations will be performed for simplicity; however, this system is not limited to single polarization but can also be extended to dual polarization. Simulation system layout parameters are listed in Table 1.

**Table 1:** Optimal phase randomization parameters.

Laser	Power	Dependent on modulation format
	Linewidth	< 0.1 MHz
PM	Phase deviation	> 70 deg
	Period	< 1024

The PM component has a phase deviation parameter, which sets the maximum phase shift applied to the optical signal. The PM phase deviation is the phase shift induced to the signal by the phase modulator. If *P*_*in*_*(t)* is the optical signal fed to the PM from the CW laser, *mod(t)* is the signal driving the PM’s electrical input, then the optical output signal *P*_*out*_*(t)* from the PM can be expressed as follows^26^:

9 $${P}_{out}\left(t\right)={P}_{in}\left(t\right)exp\left(j\times \Delta {\Phi }_{enc}\times mod\left(t\right)\right), $$

where $\Delta {\Phi }_{enc}$ represents the phase angle deviation.

Moreover, we vary another parameter for the PM called period which is defined as the number of symbols for which $\Delta {\Phi }_{enc}\times mod\left(t\right)$ remains constant in the encryption key.

System security is maximized by selecting phases between zero and the phase deviation. Furthermore, coherent state phases were randomized with a MATLAB component. A period parameter was used to set the length of bits for which a phase would remain constant. Our algorithm uses a constant period that changes the phases after a pre-defined number of bits, this period can also be randomized to further improve security and prevent Eve from determining Bob’s secret key; however, for each modulation format, a minimum period value is required at a specific transmission rate due to the BER. Unless otherwise stated, the phase deviation will be set to 90 deg, the period will be set to 1024, and 5 sets of simulations will be performed per analysis with averages and standard deviations shown. The optical signal’s power generated at both transmitter (Alice’s CW laser) and receiver (Bob’s LO) will be matched. Lastly, we assume Kerckhoff’s principle^18^ for Eve which states that the eavesdropper has the same receiver system as Bob, has an available tapping port at Alice’s output, and all information of the system but not the information related to the randomized phase pattern (period value, PRNG seed).

## Results

Various simulations are performed to determine the performance and system security:

1. CW power analysis
2. PM phase deviation and period parameter analysis
3. CW laser linewidth

Simulations were performed using OptiSystem 18 for PSK and QAM modulation formats (QPSK, 16-PSK, and 128-QAM). For test cases involving an Eve tap, it will be assumed to be “invisible” and ideal where only a maximum of 10% power can be tapped and with no coupling loss. These results demonstrate one random pattern used, however, a more detailed investigation for different random patterns can be found in^18^. When Eve taps the optical fiber, a finite optical power will be siphoned off. The variance of Δ $({\varphi }_{b}+{\varphi }_{a})$ will increase for the tapped signal for Eve and the remaining data signal for Bob. Of course, the tapped signal comes with bigger Δ $({\varphi }_{b}+{\varphi }_{a})$ and variance than the signal to Bob. Therefore, we will assume that Eve can only tap a maximum of 10% for all simulations.

### CW power analysis

**Figure 4:**
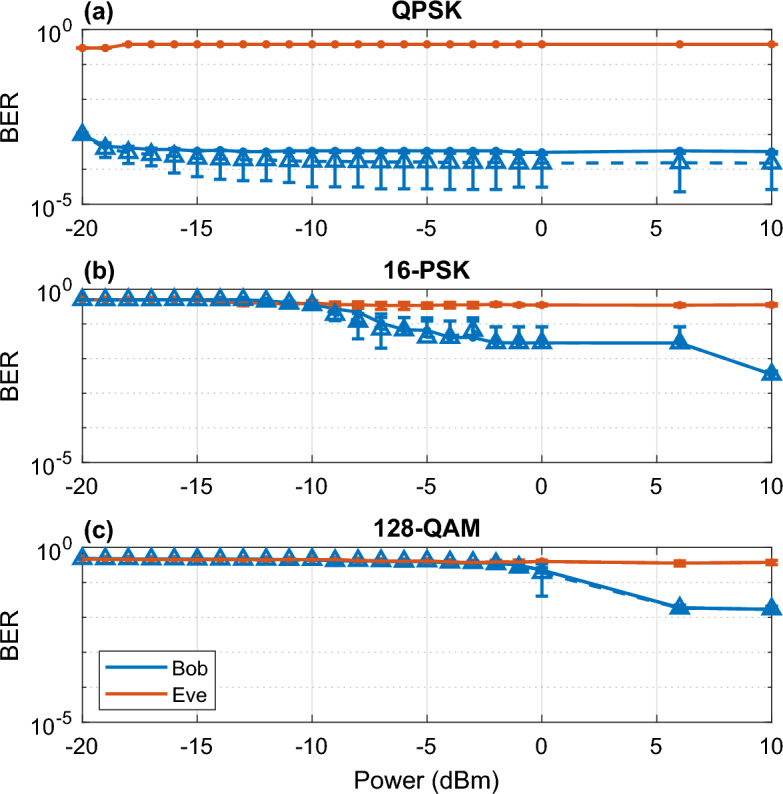
BER vs. CW laser power simulation results of receiver Bob with an attacker tapping (red line) and no tapping (blue line), and attacker Eve for (**a**) QPSK, (**b**) 16-PSK and (**c**) 128-QAM.

Figure 4 shows the simulated BER results when varying the CW power of both Alice and Eve, which is the power emitted by Alice’s laser which is represented by the “CW” block in Fig. 3. As stated above, a window of operation must be defined, where Eve is unable to determine the correct bits. For QPSK with a period of 1024 and phase deviation of 90 deg, all CW power tested can be used to obtain a BER below ${10}^{-4}$. For 16-PSK with a period of 1024 and phase deviation of 70 deg, a CW power of 7 dBm or greater is required to obtain a BER below ${10}^{-2}$. Similarly for 128-QAM with a period of 1024 and phase deviation of 90 deg, a CW power of 6 dBm or greater is required to obtain a BER near ${10}^{-2}$. Results for other modulation formats exhibited similar performance where more complex formats required working ranges at stronger input power.

**Figure 5:**
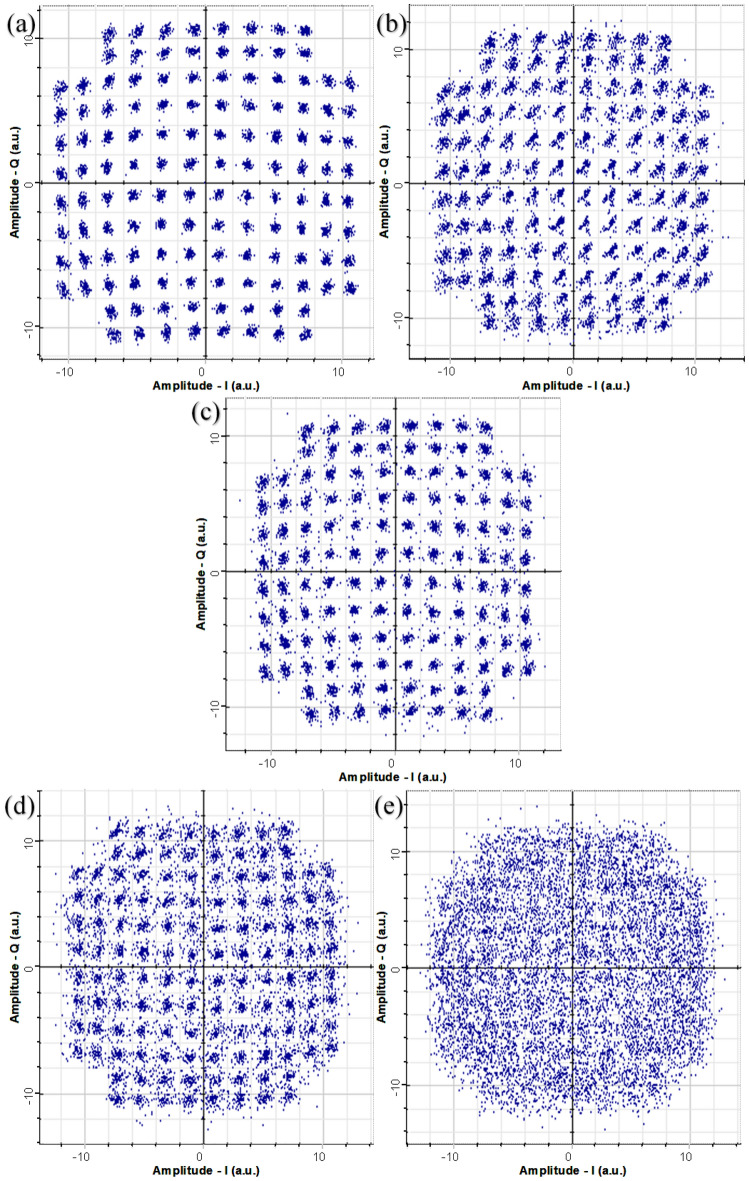
Constellation diagrams for 128-QAM without EPS for Bob with no tapping (**a**), and Eve (**b**). Constellation diagrams for 128-QAM with EPS for Bob with no tapping (**c**), Bob with tapping (**d**) and Eve (**e**).

In Fig. 5, we demonstrate the security of EPS with the initial constellation diagrams. In Fig. 5(a,b), we simulate a tapping attack with detections at Bob and Eve when no encryption at Alice is applied. Without encryption, the eavesdropper will clearly be able to obtain a relatively low BER value, allowing them to obtain information Alice sends to Bob. This means, a copy of any transmitted data, either encrypted ciphers or plaintexts, through fibers would be obtained by eavesdroppers. That is a fundamental fact for today’s optical infrastructure. However, with encryption, this capability is disabled for an eavesdropper as shown in Fig. 5(c,e). The constellation diagram that Bob receives without Eve’s tapping, is clear and the samples can be determined to the correct constellation points with minor errors. With the addition of a tapping attack, the constellations that Bob receives, are relatively good, but with noticeably more error than without a tapping attack. The constellations that Eve receives, are a randomized cluster that results in high indiscernible information with a BER of 0.5. This randomized cluster is a result of the encryption applied where each bit has been randomly shifted, changing the position, and making the constellation diagram for an eavesdropper uninterruptible. Finally, these results are in line with our experimental results which were presented in^26^ for QPSK.

### PM phase deviation and period parameter analysis

The PM phase deviation and period parameter also play a vital role in determining the security of the system. Phases for every period will be selected between zero and the phase deviation value. It was determined in^22^ that those different parameters had a large effect on the BER. In this analysis, we will perform similar performance tests as done in^22,23^ in the presence of a tapping attack. Furthermore, we will compare the BER performance with different parameters in Alice’s laser, Bob’s reference laser, and Eve’s reference laser.

**Figure 6:**
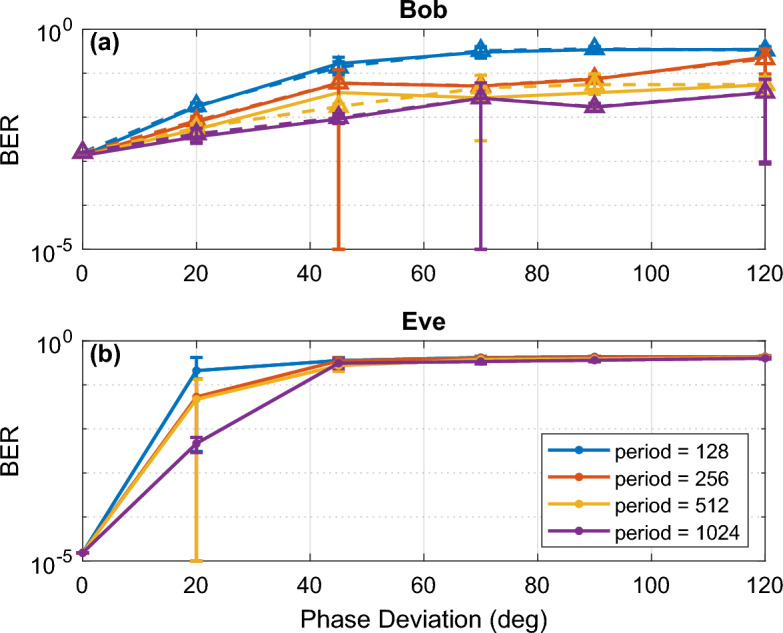
128-QAM BER vs. phase deviation simulation results of (**a**) Bob receiver with an attacker tapping (solid line), and no tapping (dashed line) for varying linewidths (LW) and (**b**) Eve for varying periods.

As shown in Fig. 6, with an increase in period, Bob’s BER decreases with a trade-off of lower security or less randomness. When the phase deviation was too small, Eve was able to obtain an error-less BER with a phase deviation of 0 deg (no phase encryption applied) and only a small error at 20 deg. A phase deviation of 20 deg resulted in low error because relatively small phase shifts were applied to the optical signal. Even without any decryption applied by Eve, with a less complex modulation format, Eve will be able to obtain a low BER for small phase deviations due to bits remaining in the correct decision boundary after encryption has been applied. At phase deviations larger than 45 deg for 128-QAM, Eve is unable to decode any information and obtains the maximum BER of 0.5.

### CW laser linewidth

**Figure 7:**
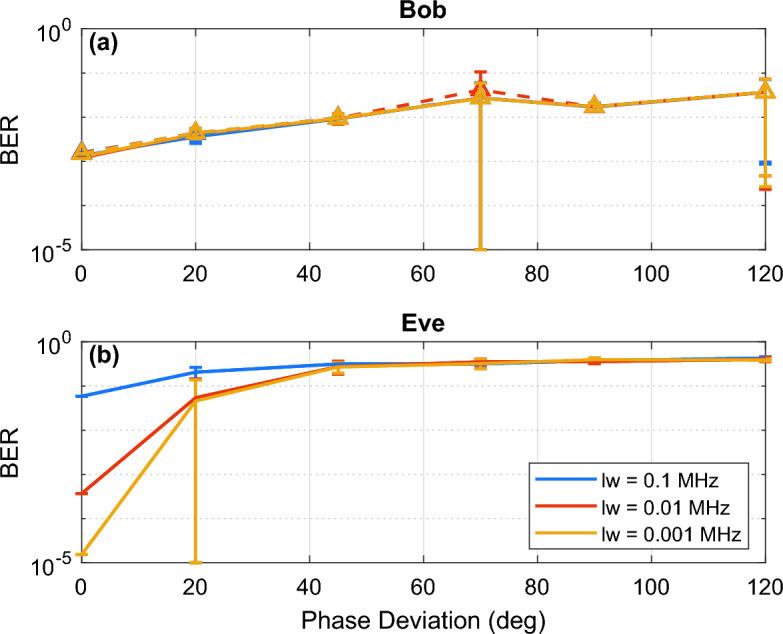
128-QAM BER vs. phase deviation simulation results at Bob receiver with an attacker tapping (solid line), and no tapping (dashed line) and (**b**) Eve for varying linewidths (LW).

Lastly, we identify the effect of the laser linewidth on the security of the system. Essentially, we determine the effect of using better laser equipment than previously tested and the effect of an eavesdropper having a superior laser than the one that Alice and Bob use in their set-up. OptiSystem is not capable of completely isolating the effects of EPS and the linewidth. At the low frequency non-linear regime instabilities may occur and may not be encompassed in this set of simulations which include white noise, flicker, random walk noises^27^. These limitations may not be a dominant effect in the present commercial network; however, with constant improvements to technology, these effects must be explored. Shown in Fig. 7 are the results for the analysis on different laser linewidth for 128-QAM. With a smaller linewidth, the BER was expected to drop significantly for Bob, though the laser linewidth was found to not improve Bob’s BER. On the other hand, for an eavesdropper, because the quality of their tapped signal and their local oscillator is improved, it was found that the security was decreased, and that Eve was able to obtain more correct bits. Nevertheless, improvements to Eve’s BER are only apparent at low to no phase deviations.

From these simulation results it was determined that a set of operating encryption parameters are required to ensure the security of the EPS system. A summary of these optimal operation parameters is shown in Table 2.

**Table 2:** Simulation test set parmeters.

Layout parameter	Sequence length	65,536 bits
	Baudrate	28 Gbaud
	PM period	1024
CW laser & local oscillator	Center wavelength	1550 nm
	Linewidth	0.1 MHz
	Azimuth	45 deg
IQ modulator	Extinction ratio	20 dB
	Switching bias	3 V
	Insertion loss	5 dB
EDFA^1^	Forward pump power	11 mW
	Forward pump wavelength	980 nm
	Loss at 1550 nm	0.1 dB/m
	Loss at 980 nm	0.15 dB/m
Optical fiber	Length (1 spool)	80 km
	Attenuation	0.2 dB/km
	Dispersion	16.75 ps/nm/km
	Dispersion slope	0.075 ps/nm^2^/km
	Differential group delay	0.2 ps/km
	Effective area	80 μm^2^

^1^OptiSystem uses the Giles and Desurvire model of the EDFA to determine gain and noise characteristics.

### Experimental results

**Figure 8:**
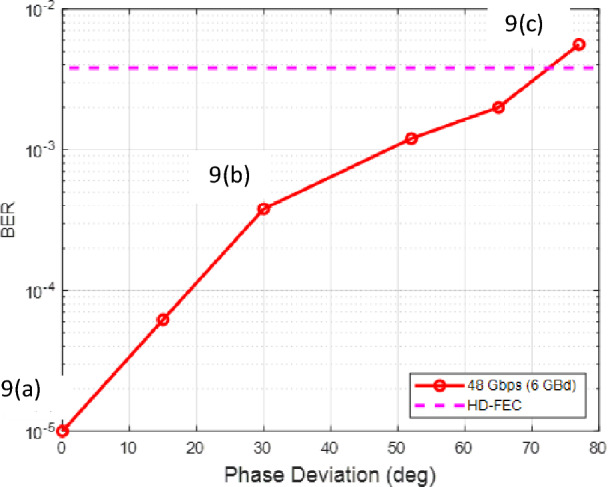
16-QAM BER vs. phase deviation experimental results of a Bob receiver with a period of 40. 9(a–c) refers to Fig. 9 constellation diagrams.

**Figure 9:**
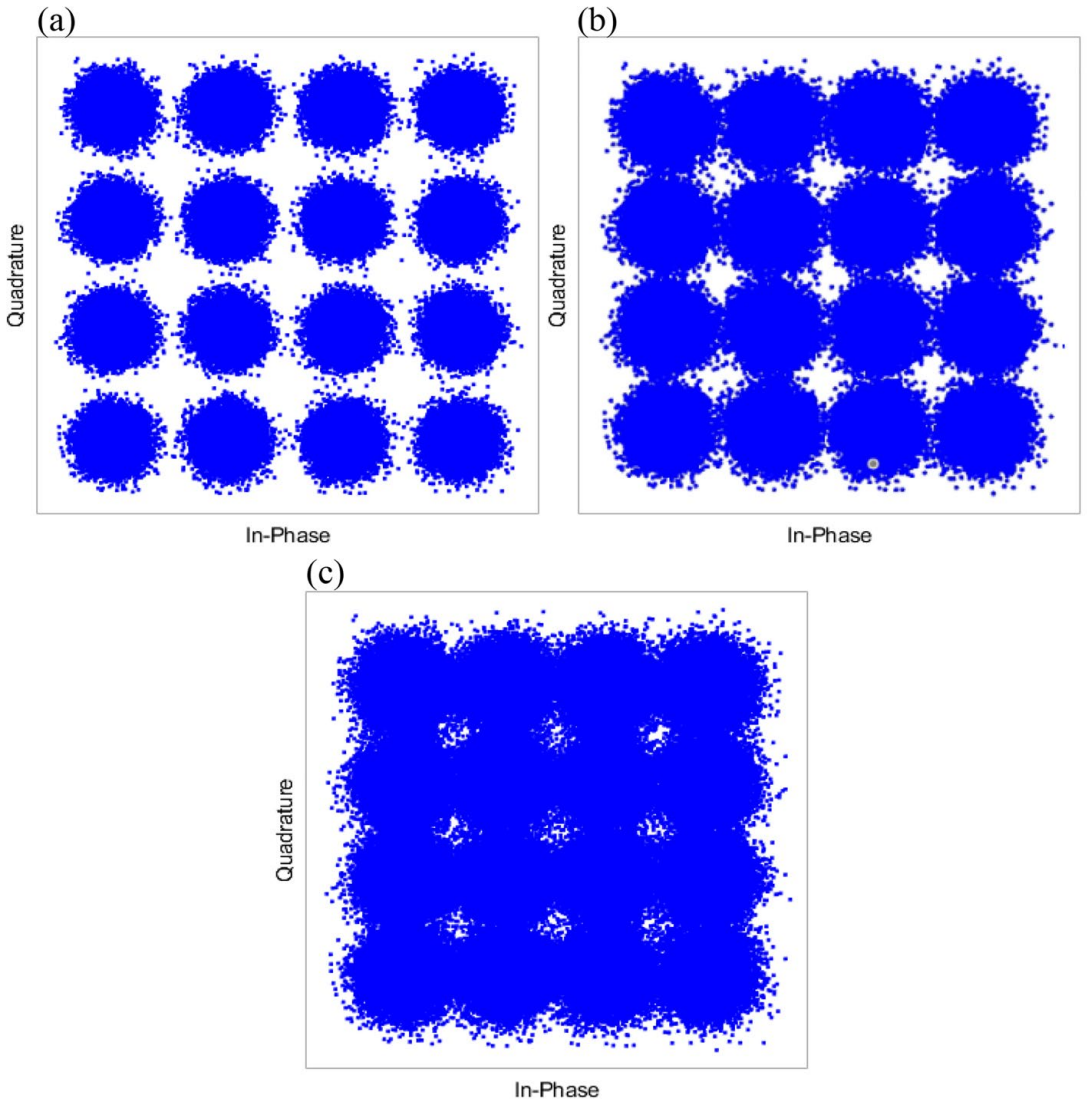
Experimental constellation diagrams for 16-QAM without EPS for Bob receiver (**a**), with EPS with a phase deviation of 30 deg (**b**) and 77 deg (**c**) for Bob receiver.

The results for an experimental demonstration are summarized in Figs. 8 and 9. The equipment and experimental setup used are similar to^26^; however, this demonstration is configured for the symmetric encryption rather than the asymmetric encryption. This highlights the configurability of the set-up allowing seamless transition from symmetric encryption to asymmetric encryption. This experiment is performed at 6 GBaud for 16-QAM. This experiment was only continued until the HD-FEC limit was reached and values differ from simulation as the experimental set-up layout parameters are not identical. As seen, the measured BER as a function of phase deviation in Fig. 8 follows the same trend as the simulations shown in Fig. 6a. With increasing phase deviation, the BER increases.

Furthermore, constellation diagrams of this experiment are shown in Fig. 9. For three phase deviation points for Bob’s receiver. To clarify, this experimental demonstration does not contain any eavesdropper and demonstrates the results for transmission from Alice to Bob transmission without any tapping. As expected, with increasing phase deviation of the encryption, the increasing BER of Bob’s receiver can be seen with the received data overlapping in constellation points. With increasing phase deviation, larger error occurs due to bigger phase shifts resulting in a larger BER which is seen in Fig. 9c where the constellation points start to overlap. It is also important to note that this is a preliminary experimental validation where future work will be performed to verify the conclusions made in this simulation at higher baud rates, more complex modulation formats, and in the presence of Eve. Nevertheless, these results verify the conclusions made for a non tapping scenario.

## Discussion

In our previous analysis^22^, we determined that by controlling the CW power of both Bob Tx and Rx, a window is created where Bob can recover Alice’s secret key while preventing Eve from obtaining any transmitted information. Furthermore, we performed an analysis on these results in^23^, determining the theoretical and practical security of the system. We have also tested for varying CW power and concluded that Eve is unable to decode for all CW power for all modulation formats, resulting in a BER value of 0.5 at all input power. These results demonstrate that EPS is compatible with all modulation formats and maintains security by encryption of the encoded data at the transmitter preventing any eavesdropper from obtaining any information. The initial encryption will fully mask Alice’s transmission data and can only be decoded with knowledge of the exact phases applied. These results demonstrate the efficacy of applying phase encryption to the security of the network in preventing any information from being known to an eavesdropper. In contrast to our results in^22^, which described a window of power operation that prevented an eavesdropper from obtaining information, in this system a minimum power is defined to provide security where even at high powers, an eavesdropper is unable to obtain any information. A minimum power is required in EPS to obtain good BER due to noise from transmission. Additionally, by operating at higher power, lower BER at longer transmission distances can be achieved. It is also important to note that for QPSK modulation, the minimum power required is outside the range of testing shown and was determined to be at a CW power greater than -21 dBm. Although the working range provides a minimum power that the operator must operate at, the BER can also be used to verify if an attacker is presently eavesdropping. In this case, only one specific power can be used, where Bob’s BER would increase significantly in the presence of an eavesdropper tapping 10% of their power (for 16-PSK, a power of -5 dBm and for 128-QAM, a power of 0 dBm). For QPSK, this case would not be possible at any power because there is no significant difference between the BER in the no tapping and tapping case. This is due to the already low operating power that EPS can operate at for QPSK.

Constellation diagrams were also shown in Fig. 9 to demonstrate the effect that EPS has on both Bob’s and Eve’s diagram. Here, the effects of adding encryption to a traditional optical network are compared. Without encryption, it was determined that Eve could obtain information sent between Alice to Bob assuming that Eve had information of their systems. With encryption, the in-phase and quadrature electrical signals at the Rx are shifted and scrambled, preventing correct detection of bits by Eve. This demonstration is extremely important to the security of EPS. By changing the phase space of the in-phase and quadrature signals of the data, malicious parties would obtain data that is unidentifiable. The bits of data are mapped to a modulation format which correlates to a constellation point and these constellation points are on the in-phase and quadrature axis. If the phase reference is shifted continuously and without any knowledge of the new phase references, a malicious party would obtain incoherent data.

We have also shown that the phase deviation and period results demonstrated that selecting large phase deviations can provide greater security in securing Alice’s data to Bob. It was also found that Eve could decode the signal up to a maximum of 45 deg. Therefore, it is recommended to use a phase deviation of at least 70 deg for any modulation format tested to prevent an attacker from obtaining any information. An analysis on Eve’s phase de-randomization in the DSP was not performed as it has been demonstrated in^28^ for various test cases and will be assumed to be similar and comparable in performance when adapted to the EPS system. Our exploration of transmission impairments on the DSP’s ability to distinguish the phase modulation from the encryption process is incomplete and will be performed in the future. This will require more time to test and verify by analyzing factors such as the effect of equalization enhanced phase noise on the DSP’s ability to properly perform decryption due to the non-commutative property of the convolution and multiplication. Optimization of DSP algorithms may be required, or new algorithms will need to be implemented to compensate for the additional encryption introduced by EPS. Nevertheless, shown in our experimental results, current DSP algorithms are capable, but not optimized to decode EPS. It is also important to note that in our simulation results large standard deviations are present within testing. These results are mainly due to one extreme outlier within the test set with a substantial increase in BER. The increase in BER shifts the average up slightly from the expected value and results in a large standard deviation. Furthermore, the logarithmic scaling amplifies these outliers, appearing more significant. Finally, it was concluded in the laser linewidth analysis, that with lower linewidth, results do not improve for an attacker when EPS is present, however, when no encryption is applied, the results obtained by an eavesdropper will be superior. As expected, with a better-quality signal, where the linewidth is improved, an attacker can obtain a lower BER after tapping. However, with the addition of EPS to the system, the phase space of the coherent state is scrambled, preventing the eavesdropper from obtaining any significant information even if the quality of their tap is improved. Thus, it can be concluded that linewidth and EPS are independent of each other; if the eavesdropper has knowledge of the EPS applied, their results will be better at lower linewidth, but if they have no knowledge of the EPS applied, their results will have no difference at any linewidth. Based on these results and summarized in Table 2, by selecting a smaller PM period value and a larger phase deviation value, the security can be maintained. Again, these results demonstrate the security of applying an encryption to prevent an eavesdropper from obtaining information sent over the optical network using different parameters.

Besides the theoretical verified security, the practical security of EPS must be considered. An eavesdropper has one main challenge in obtaining the secret information sent from Alice to Bob due to the phase randomization pattern. Eve can attempt to decrypt the encoded data through their own PM or through digital decryption, however, without knowledge of the pre-shared seed, Eve will be unable to obtain the correct bits. Although slightly simpler for EPS, where the attacker can also decode and compensate digitally, the obstacle of having the correct random seed remains. With a cryptographic-secure PQRNG^24^, the sufficiently large entropy will deter attackers from attempting to decrypt the encryption even when part of the initial or running state becomes available due to future states being unpredictable. The said PQRNG is capable to take a secret of up to 16 kB long, perfect for EPS. Moreover, the complexity of this system can quickly be increased through randomizing the phase deviation and period parameter during operation. This additional randomization must be driven by a deterministic PRNG component so that encryption and decryption can be seamless. It is recommended that the same PRNG unit used to drive the PM should be used to randomize the phase deviation and period parameter to reduce resources. Furthermore, tested in both simulation and experiment previously, if there is even one symbol shifted in the phase de-randomization, then a maximum BER of 0.5 is obtained. This is similar to^23^ which discusses how synchronization is a monumental challenge for an eavesdropper in decoding.

Finally, we briefly discuss other forms of physical layer attacks on the system. Common vulnerabilities in optical networks that can still be exploited such as gain competition in erbium-doped fiber amplifiers, interchannel crosstalk, correlated jamming, and denial of service through fiber damage^29^. All of these disruptions can cause major issues to the optical network service; however, the network security and robustness can be maintained through traditional network routing algorithms^29^, minimizing system disruptions. EPS can be integrated into current infrastructure allowing it to leverage these technologies. With the vast array of interconnected optical fiber, service disruptions can be quickly rerouted to reduce physical impairment. With the addition of EPS to current infrastructure, there is little to no added complexity with the advantage of increased security at the optical layer. Only one optical component is added to the system for the encoding and decoding can be performed in DSP. Thus, EPS can be used as a physical layer security against attacks where the malicious party target the data in the network, however, for attacks which are targeted to disrupt a network, current solutions can be still leveraged.

## Summary

We have proposed a theoretical model for an optical encryption layer scheme over existing coherent optical communication channel utilizing randomized phase encoding. EPS is compatible with common modulation formats such as PSK and QAM. Both PSK and QAM formats were tested at 28 GBaud. A common eavesdropping scenario was considered, and it was demonstrated that the system was secure against an eavesdropper with no knowledge of the randomization seed. With larger phase deviation and smaller periods, the security of the system increases with a trade-off of higher BER. In contrast to^20^, it was concluded in the CW power analysis that a minimum power was required for operation instead of a window of operation range. Constellation diagrams were also compared to demonstrate the effect of encryption has only the optical network. Preliminary experimental validation was performed, and results were in line with simulation results. Other physical layer attacks were also discussed where the addition of EPS does not add vulnerability to currently used conventional coherent optical communication networks, however, EPS can still be affected by common disruptions. EPS provides a unique solution to telco operators to control the data security of their system over the infrastructure layer. This model will be employed experimentally to validate our numerical results and is the subject of current study.

## Data Availability

Data are available from the corresponding author upon reasonable request.
